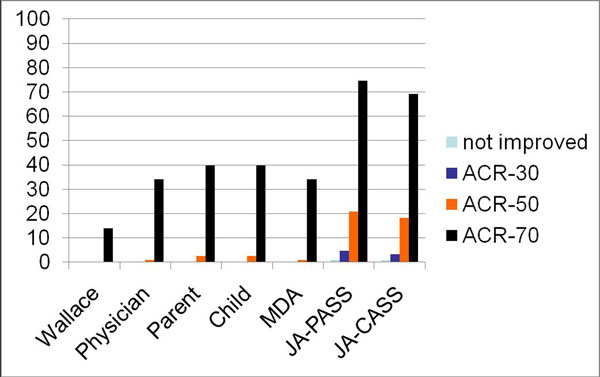# A new approach to defining inactive disease, minimal disease activity and parent- and child-acceptable symptom state in juvenile idiopathic arthritis

**DOI:** 10.1186/1546-0096-9-S1-P196

**Published:** 2011-09-14

**Authors:** A Consolaro, G Filocamo, M Bertamino, E Palmisani, G Bracciolini, C Suffia, V Muratore, N Ruperto, A Martini, A Ravelli

**Affiliations:** 1 Dipartimento di Pediatria, Università degli Studi di Genova, Italy; 2 Pediatria II, IRCCS G.Gaslini, Genova, Italy; 3 Dipartimento di Pediatria, Ospedale S.Orsola, Bologna, Italy; 4 Clinica Pediatrica, Fondazione IRCCS Policlinico S. Matteo, Pavia, Italy

## Background

Recent advances in treatment of juvenile idiopathic arthritis (JIA) have markedly increased the potential for achieving a remission or minimal disease activity (MDA) state. So far, these states have been defined using a categorical model, which requires simultaneous fulfillment of multiple criteria. However, a dimensional model, based on pooling individual measures into a composite score, has the advantage of providing one summary number on a continuous scale.

## Aim

To develop and validate the cut-point values of the Juvenile Arthritis Disease Activity Score (JADAS)-10 for remission, MDA and parent/child acceptable symptom state.

## Methods

480 children with JIA who underwent 1218 visits were included. At each visit, the physician, a parent and the child were asked to rate subjectively the disease status as remission or active disease. Furthermore, the parent and the child were asked to declare independently whether they were satisfied or not satisfied with the current illness state. All visits in which the inactive disease (ID) criteria or MDA criteria were met were identified. The “optimal” JADAS cut-point for each disease state was determined through the ROC curve analysis. To minimize misclassification, the minimum specificity of 85% was required. Validation of cut-points was carried out by assessing their ability to discriminate between different levels of ACR Pediatric response in a methotrexate trial and between different levels of damage, assessed through the Juvenile Arthritis Disease Activity Index (JADI).

## Results

**Figure 1:**
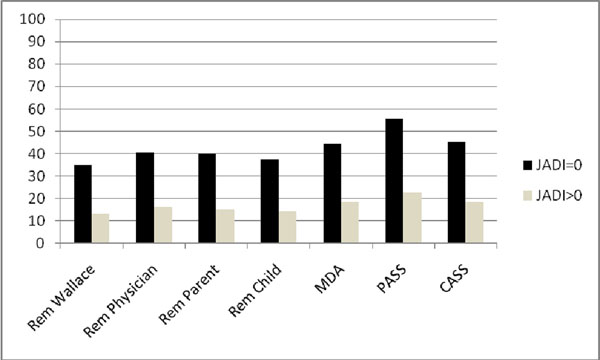


The JADAS-10 cut-points for ID, MDA, physician-, parent- and child-rated remission, parent and child acceptable symptom state were 2, 3.8, 2.9, 2.6, 2.0, 3.8, 5.3, and 3.1 for polyarthritis and 1, 2.5, 2.5, 2.3, 2.2, 4.5, and 3.1 for oligoarthritis. Assessment of discriminative ability of cut-points is shown in Figures 1 and 2, respectively.

## Conclusion

JADAS cut-points for ID were the lowest, which reflects the greatest stringency of Wallace criteria. Cut-points for children’s assessments were lower than for physicians’ and parents’ assessments, suggesting that children require a better disease control to consider the disease in remission or declare themselves as satisfied. All JADAS cut-points discriminated strongly between different levels of therapeutic response and damage, which indicates that they are suitable for use in clinical practice, observational studies and clinical trials.

**Figure 2:**